# Book Reviews

**Published:** 1802-07

**Authors:** 


### CRITICAL RETROSPECT OF MEDICAL AND PHYSICAL LITERATURE.

*The Report made to the National Inftitute of France, in the Month of December* 1799, *by Citizens Portal, Pelletan, Fourcroy, Chaptal, and Vauquelin, refpecting the Artificial Mineral Waters, prepared at Paris by* NICOLAS PAUL *and Co. with Extracts from the Reports of the Society of Phyficians of Paris, and the Faculty of Geneva; and other Teftimonies in Favour of the fame Waters: To which are added, fome Notes and Obfervations, by* N. PAUL. 8*vo*, *pp*. 64. London, 1802, Woodfall.

In the Dedication to Count Rumford, Mr. Paul thus expreffes himfelf,

"Confident of the intereft you take in every thing that relates to the benefit of mankind, I venture to claim your patronage to an undertaking which has for its object a concern equally important and univerfal, the Prefervation of Health, and the Relief of Difeafe. It is to no fanciful untried fcheme, Sir, that I folicit your attention, but to an extenfive and ufeful improvement of chemical difcoveries, which had their rife in England, and which by unceafing efforts, and by the liberal fupport of enlightened men, I have happily fucceeded in bringing to a greater degree of matutiry in a foreign country.

"Many

"Many years have elapfed, fince I firft engaged in a manufacture of Artificial Mineral Waters at Geneva. The diftinguifhed approbation which that undertaking met with from the phyficians of that place, and from the public at large, induced me to form another, and ftill more extenfive one, of the fame kind, at Paris. This, Sir, you have, not long ago, been pleafed to vifit; and the annexed reports refpecting it, which are drawn up by Committees felected from amongft the ableft chemifts and phyficians in France, will concur with your teftimony, to fhow that its claims to encouragement are not founded upon myfterious fecrecy or fpecious pretence, but reft upon the firm bafis of fcientific examination and approved ufe.

"The characteriftic liberality of the Britifh Nation, entitles it, in an eminent degree, to fhare in the advantages of whatever is calculated for the good of fociety; but it has a peculiar claim to benefits derived from the application of difcoveries which originated from itfelf."

In his Preface, the Author explains his views more particularly:

"Having been ftrongly encouraged, by fome friends in this country, to eftablifh in London a laboratory, or manufacture of artificial mineral waters, upon the new plan, and with the feveral improvements which I have introduced abroad into that department of practical chemiftry, I have lately repaired to England for that purpofe; and although not quite a ftranger in this city, where I refided about fifteen months at a former period, I thought my firft care ought to be to convey to the public an idea of my object, and what I confider as my claims to its fupport. In this view I have been induced to publifh the annexed Report made to the National Inftitute of France by a felect committee of eminent medical men and chemifts, refpecting the manufacture of mineral waters and the bathing eftablifhment, which, within thefe two years, I have fet on foot at Paris. This Report, I have reafon to fuppofe, will attract the public attention more effectually than any account or commentary of my own. It will fhow, that not only phyficians and men of fcience abroad have thought it worthy of their attention to inquire minutely into this eftablifhment, but that it has, in fome degree, become in France an object of public concern.

"To this Report I have only fubjoined, in the form of an appendix, a few notes, the principal of which contain teftimonies from the Faculty of Paris, and from the phyficians of Geneva. To the latter efpecially, who have never ceafed to give me all poffible encouragement and affiftance, I feel great pleafure in expreffing, upon this, as upon every occafion, my gratitude and regard.

"My intention, at prefent, is to confine myfelf to the preparation of all fuch kinds of mineral waters as are either taken medicinally, or drank as a luxury, and as a falubrious article of diet, laying afide the bathing eftablifhment, and all that relates to the *external* ufe of mineral waters. But the apparatus and laboratory are equally calculated to prepare any kind, or any quantities of mineral waters, that may be required for any purpofe whatever.

"The public will now be conftantly and regularly fupplied with any of the mineral waters that are mentioned in the annexed reports, and and with ſuch other kinds, or ſuch new modifications of thoſe waters, as phyſicians may think proper to ſuggeſt.

"The mineral waters that will be kept immediately in readineſs are:

"1. The ſtrong Seltzer Water.

"2. The mild Seltzer Water.

"3. The ſtrong Spa Water.

"4. The weak Spa Water.

"5. The Gaſeous Alkaline Water (commonly called *mephitic*) either with carbonat of ſoda or carbonat of pot-aſh.

"6. The Seidlitz Water.

"7. The Oxygenated Water.

"8. The Hydro-carbonated Water.

"9. The Hydro-ſulphurated or Hepatic Water.

"I do not know how far the uſe of mineral waters has been introduced into the hoſpitals of this country; but I conceive that they may, in ſome inſtances, ſuperſede the uſe of wine or other expenſive articles. I ſhall be happy, if this ſhould be found to be the caſe, to ſupply public charitable inſtitutions, on any terms that their general welfare may be thought to require."

---

*Obſervations on the Arguments of Profeſſor* Rush, *in favour of the Inflammatory Nature of the Diſease produced by the Bite of a Mad Dog. By* James Mease, M. D. *8vo. pp.* 64. *Philadelphia,* 1801.

The candid, liberal, and philoſophical manner in which Dr. M' has examined, and, as we think, refuted the arguments of the learned Profeſſor, may ſerve as a model well deſerving the imitation of Medical Controverſaliſts. The man is every where kept out of ſight, his reaſoning alone being preſented to our view. When Dr. M. comes to deliver his own method or plan of treating hydrophobia, he propoſes to exhibit wine, opium, &c. in much larger doſes than practitioners have been in the habit of employing.

"In my Inaugural Eſſay, I recommended the uſe of opium, on the principle of its anti-ſpaſmodic virtue, but in much larger doſes than it had ever been preſcribed in the diſeaſe, becauſe I perceived that the ſmall quantities which had always been preſcribed, never in the leaſt mitigated the ſymptoms. But from the examples before ſtated of its inefficacy, even when given in larger doſes than I thought the ſyſtem could bear, I am now convinced, that it is loſing time to truſt to it alone. In its place I would recommend the uſe of the powdered leaves of Stramonium (Thorn apple, or James Town weed) or their extract, in doſes of two grains for an adult. By that quantity Dr. Cooper (Inaug. Diſſert. 1797) found the pulſe "increaſed in frequency at firſt, and that it afterwards became full and quick, produced giddineſs, *warm skin*, moiſt hands, and *sleepineſs*." A defect of due energy in the heart, wakefulneſs, and cold ſkin, are ſymptoms that conſtantly attend the diſeaſe, and the two laſt are the ſources of much diſtreſs. Hitherto no no remedy has had the leaft effect in removing them. Their cure will greatly affift toward the removal of the whole complaint. This may be effected in my opinion by the *stramonium*, if given *early* in the difeafe. It fhould be exhibited in fuch dofes as will *powerfully affect the system*, and repeated as often as a previous dofe has ceafed to act. During the fufpenfion of the fymptoms, bark and wine ought to be given, and the dofe gradually increafed, fo as to keep up a regular excitement and produce a permanent vigour in the fyftem. The quantity of wine may be unlimited. Indeed, the only rule that ought to be obferved with refpect to it, is, to *give it in as large quantities as the stomach will bear, and until it produces the desired effect*. For this difeafe exhibits a fingular inftance in the concentration of fenfibility in certain parts of the body, and of a great defect of it, nay almoft a total exhauftion of it in another. We fee the fame thing in tetanus and other difeafes. Thus, while the eye cannot bear the fight of a looking glafs, or a vivid colour; nor the ear the fhutting of a door, nor the fkin nor lungs the impreffion of the air, the ftomach is fo infenfible to the impreffion of ftimuli, that a bottle of wine will not produce as much effect on the pulfe as a few glaffes will in times of health. Dr. Currie of Liverpool cured a cafe of tetanus by one hundred and ten bottles of wine, and obferves, that " ebriety was not produced; it foothed the irritation of the nerves, and comforted the mind, and without increafing the frequency of the pulfe, it augmented it in ftrength*."

" In cafe however the above remedies cannot be obtained or exhibited; I fhould have no hefitation in trying another plan which has feveral arguments to authorize the experiment, although at firft view it may appear to be attended with danger. It is, *to excite a stranguary by means of Cantharides*.

" The principle of the animal œconomy, firft unfolded by J. Hunter, of one irritation curing another, is daily and amply confirmed in practice, and its application in the prefent difeafe feems highly probable. Without referring to the many inftances afforded, in illuftration, I may adduce one difeafe which is nearly allied to the prefent, viz. tetanus. When this is occafioned by the lefion of a nerve from a rufty nail, or other pointed inftrument, we find it readily yields to an irritation of the wounded part, raifed by fcarification and the application of hot turpentine, marine falt, or cantharides; and in the progrefs of the difeafe, or when it fucceeds the expofure of the body to dews and night air after being heated in

* The defect of fenfibility exhibited by perfons labouring under fome difeafes of debility is really aftonifhing. A delicate young lady of Philadelphia, a few years fince, was recovered from the loweft ftate of a tyhphus or low fever, by the ufe of 127 bottles of old Madeira, which was given to her, at her own requeft, when fo weak, that fhe could fcarcely be heard to pronounce the fingle word " *wine*." From one table fpoonful fhe took at laft, two bottles a day. I have alfo feen children in the *cholera*, or fummer complaint, bear almoft incredible dofes of ftimulants.

in ſummer, an irritation of the ſalivary glands by mercury as readily proves effectual. A knowledge of theſe facts, and a conviction of the truth of the principle, would have been ſufficient to prevent my heſitating to try the plan I propoſe, but I am now confirmed of its utility and perfect ſafety, in conſequence of the cure by its uſe, of a deſperate caſe of tetanus, by Dr. S. Brown of Lexington Kentucky, who lately communicated the hiſtory to Dr. Ruſh. The patient, a lady, was nearly exhauſted by the diſeaſe, when her judicious phyſician gave her the tincture of cantharides, which by exciting a temporary inflammation in the ſtomach and bowels, and producing a ſtranguary, effected a cure. The moſt dangerous pleuriſies have alſo been cured by the late Dr. Lieper of Maryland, after the common remedies had failed, by exciting a ſtranguary by means of the ſame tincture mixed with camphorated ſpirit of wine; and when combined with tincture of Peruv. bark, and given with the ſame view, it has been recommended by experience, in the hooping cough.

"The recommendation of the remedy in this diſeaſe produced by the bite of a mad dog is not new. Morgagni mentions its general uſe for the cure of the diſeaſe in Germany; his remark is confirmed by a late author. A Sileſian peaſant alſo acquired much reputation for the cure of the diſeaſe; and on the purchaſe of his ſecret by the King of Pruſſia, in 1777, the baſis was diſcovered to be the *meloe proscarabæus et majalis* (oil beetle.) All the inſects of the meloe tribe poſſeſs a bliſtering quality. In a diſeaſe which has hitherto ſo generally proved ſuperior to all the efforts of medicine, it is a duty to try every plan which promiſes the leaſt ſucceſs. The one I now urge, is ſupported by a juſt theory, a cloſe analogy, and if we admit the German authority, I may add, is proved by experience."

---

*An Inquiry into the Modus Operandi of Medicines upon the Human Body. To which are added, some Observations on the Action of the Lymphatics. By* WILLIAM WYATT BIBB, *of Georgia, Member of the Philadelphia Medical and Chemical Societies. pp.* 68. *Philadelphia.* 1801.

THE author of this Eſſay endeavours to prove, "that all medicines act ſpecifically * upon one or more parts of the body; that they are all ſtimulants †; and that their various effects, which have induced writers to divide the materia medica in ſtimulants, tonics, aſtringents, &c. ariſe entirely from a ſtimulant *specific* operation, exerted upon their appropriate parts of the animal ſyſtem.

"Believing

---

* As the word *specific* is frequently employed in the following pages, and as it has often been uſed indefinitely, it is neceſſary I ſhould define what is meant by it. I mean then, that every medicine operates in a way peculiar to itſelf, and acts, in whatever manner applied, on particular parts chiefly.

† In aſſerting that all medicines are ſtimulants, I only include medicines properly ſo called. The abſtraction of ſtimuli from the body, is not conſidered as entitled to that application.

"Believing every difeafe, to which the body is fubject, to have a particular feat, and every medicine to exert a *specific* operation, I think myfelf fupported by the laws of nature, in dividing the general fyftem into feveral leffer ones. Directed then by the effects of difeafes, and the operation of medicines, I fhall divide the body into fix fyftems, viz. 1ft. The Vifceral Syftem, in which I include the ftomach and inteftines. 2d. The Sanguiferous Syftem. 3d. The Nervous Syftem, including the brain and nerves. 4th. The Mufcular. 5th. The Abforbent; and, 6th. The Glandular Syftem.

"The relation which thefe different fyftems bear to each other is very unequal. The connection of the ftomach with all of them is far more intimate than that which fubfifts between any others. The different parts of the body, therefore, are very liable to be affected, through the medium of this vifcus, both by difeafes and medicines, and vice verfa."

He then proceeds to examine the feveral kinds of medicines which act upon each of thefe fyftems, and confirms his reafoning by experiments made on himfelf and friends: from all which he infers, that he is "fupported by truth, in deducing the following conclufions.

"1ft. The different difeafes of each fyftem are curable, or incurable, according to the progrefs which has been made in difcovering their *specifics*.

"2d. The only method by which we can arrive at certainty, in curing difeafes, is, by attentively inveftigating the operation of medicines upon the feparate fyftems of the body.

"The doctrine of *specifics* which I have delivered, leads to the moft important objects in the practice of medicine. 1ft. To a proper felection of remedies for curing the difeafes of every fyftem, whereby we are capable of acting upon the difeafed part, without much affecting the healthy ones. 2d. To a proper felection of medicines, to excite new action in parts lefs effential to life, thereby abftracting morbid action from vital ones. Thefe then being objects of the greateft magnitude, it is our duty to adopt every confideration which may, in the fmalleft degree, promote their accomplifhment. Nothing, in my opinion, will fo certainly produce this effect as a well directed attention to the partial operation of medicines, and a judicious arrangement of the different articles of the materia medica according to the fyftems, or parts of fyftems, upon which they are found to operate. The advantages that would refult from fuch an arrangement, over every other hitherto propofed, are many and important.

"1ft. According to the hitherto adopted divifion into ftimulants, aftringents, tonics, &c. we are incapable of felecting with any degree of certainty fuch medicines as are adapted to the cure of the difeafes of a particular fyftem. Is a patient labouring under tetanus? It is a common, and I believe a proper practice, to employ ftimulants in moft cafes. Now, by what are we to be governed in our felection from this numerous clafs? By nothing, as far as I know, but the peculiar whim of the phyfician; hence has arifen the exhibition of fuch

ſuch a vaſt variety of appropriate medicines for the cure of every diſeaſe.

"But, according to the arrangement I have propoſed, we have fixed laws to direct us. The firſt circumſtance to be aſcertained, before we preſcribe for a patient, is the ſyſtem or ſyſtems diſeaſed, and the nature and grade of morbid action; then by referring to that claſs of medicines which is appropriated for the diſeaſes of thoſe parts, which are now affected, we can at once ſelect the proper remedies.

"2d. A proper examination of the operation of medicines upon the ſeparate ſyſtems, is the only proper method of aſcertaining their properties. If, in exhibiting them, we direct our attention only to their effects upon the whole ſyſtem as one indiviſible machine, their operation upon the different parts may eaſily paſs unnoticed; but by minutely obſerving their action upon the ſeparate ſyſtems, we ſhall diſcover the parts upon which they act ſpecifically, and profit accordingly."

